# Supplementary material for: Human Adipose Mesenchymal Stromal/Stem Cells Improve Fat Transplantation Performance
Source: Cells. 2022 Sep 7;11(18):2799. doi: 10.3390/cells11182799 (PMC9496721; doi:10.3390/cells11182799)
Supplement: Supplementary file 1 [file cells-11-02799-s001.zip › cells-1848301-supplementary.pdf]

cells

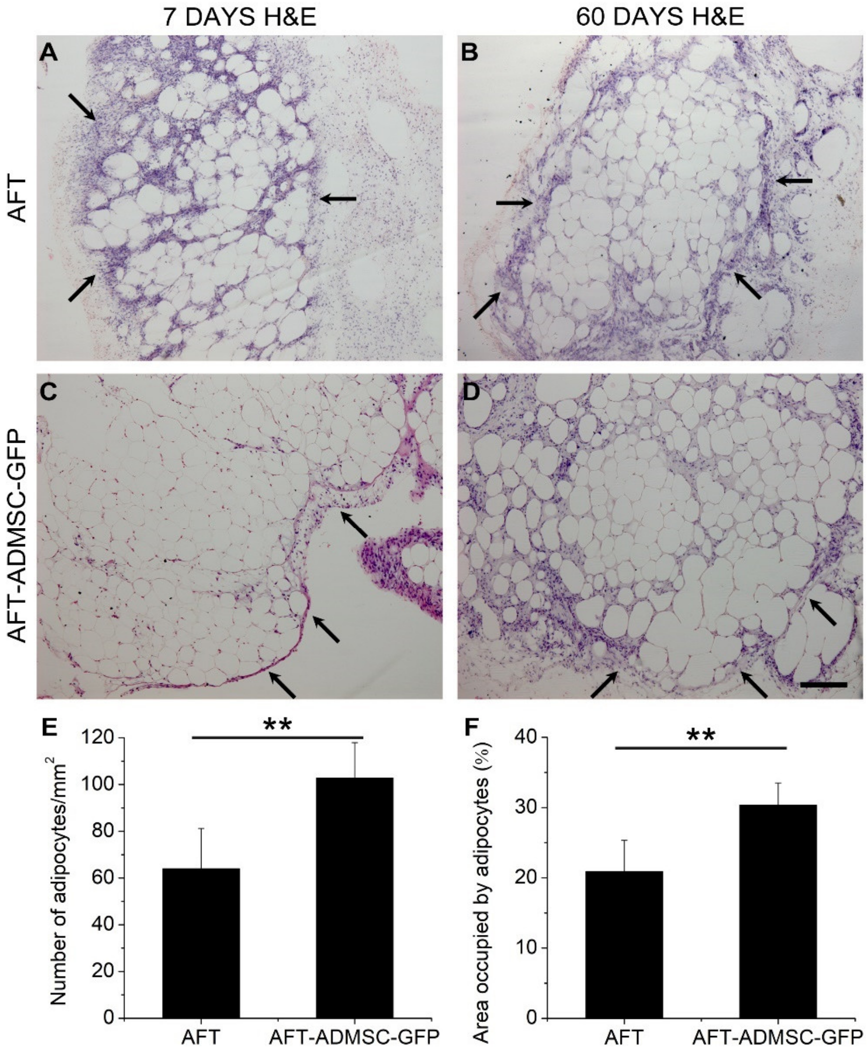

Figure S1. H&E staining of a thin layer of connective tissue in the external area of the explants and adipose tissue characterization: (**A**) AFT graft at 7 days post-transplantation; (**B**) AFT graft at 60 days post-transplantation; (**C**) AFT-AD-MSC-GFP graft at 7 days post-transplantation; (**D**) AFT-AD-MSC-GFP graft at 60 days post-transplantation; connective capsulae are indicated by black arrows; (**E**) average number of adipocytes per area unit (mm^2); (**F**) percentage of adipocytes per area in the group. Images were acquired using an AxioZoom V16 (Zeiss) at a magnification of 270×. Scale bar = 200 µm; ** p-value < 0.01.

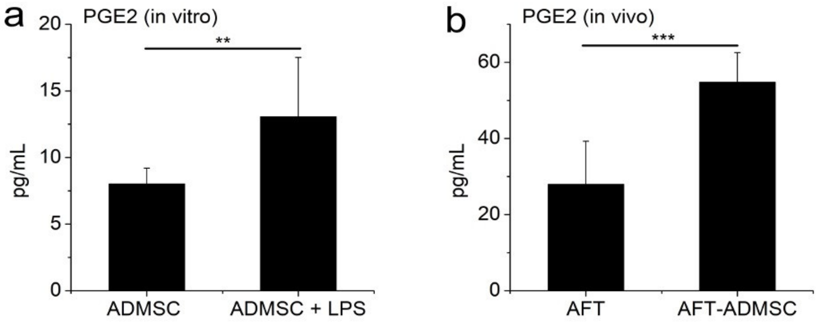

Figure S2. PGE2 quantification by in vitro and in vivo assays: (**a**) AD-MSCs were stimulated for 24 h by LPS (8 µg/mL) in vitro (n = 3), and supernatants were collected and analyzed by ELISA, ** p-value < 0.01; (**b**) mice sera were collected from the AFT and AFT-AD-MSC groups at 180 days after transplantation, and PGE2 levels were quantified by ELISA, *** p-value < 0.001.
